# *Bassanianabirudis* sp. nov., a new crab spider (Araneae, Thomisidae) from South Korea

**DOI:** 10.3897/BDJ.9.e73109

**Published:** 2021-11-17

**Authors:** Jae Seong Im, Seung Tae Kim, Sue Yeon Lee

**Affiliations:** 1 Entomology Program, Department of Agricultural Biotechnology, Seoul National University, Seoul, Republic of Korea Entomology Program, Department of Agricultural Biotechnology, Seoul National University Seoul Republic of Korea; 2 Life and Environment Research Institute, Konkuk University, Seoul, Republic of Korea Life and Environment Research Institute, Konkuk University Seoul Republic of Korea; 3 College of Agricultural Life Science, Jeonbuk National University, Jeonju-si, Republic of Korea College of Agricultural Life Science, Jeonbuk National University Jeonju-si Republic of Korea

**Keywords:** Thomisidae, *Bassaniana*, new species, description, South Korea

## Abstract

**Background:**

The crab spider genus *Bassaniana* Strand, 1928 consists of six species mainly distributed in North America and Far East Asia. Two species of them, *Bassanianadecorata* (Karsch, 1879) and *Bassanianaora* Seo, 1992, are known in Korea so far.

**New information:**

A new crab spider, *Bassanianabirudis* **sp. nov.** is described, based on a male collected from Gumi-si, Gyeongsangbuk-do, South Korea. Distribution records are provided, as well as photos of habitus and illustrations of the male copulatory organ. The type specimens of this study are deposited in the collection of the Nakdonggang National Institute of Biological Resources (NNIBR) and Konkuk University (KKU), South Korea.

## Introduction

**Figure 1.**
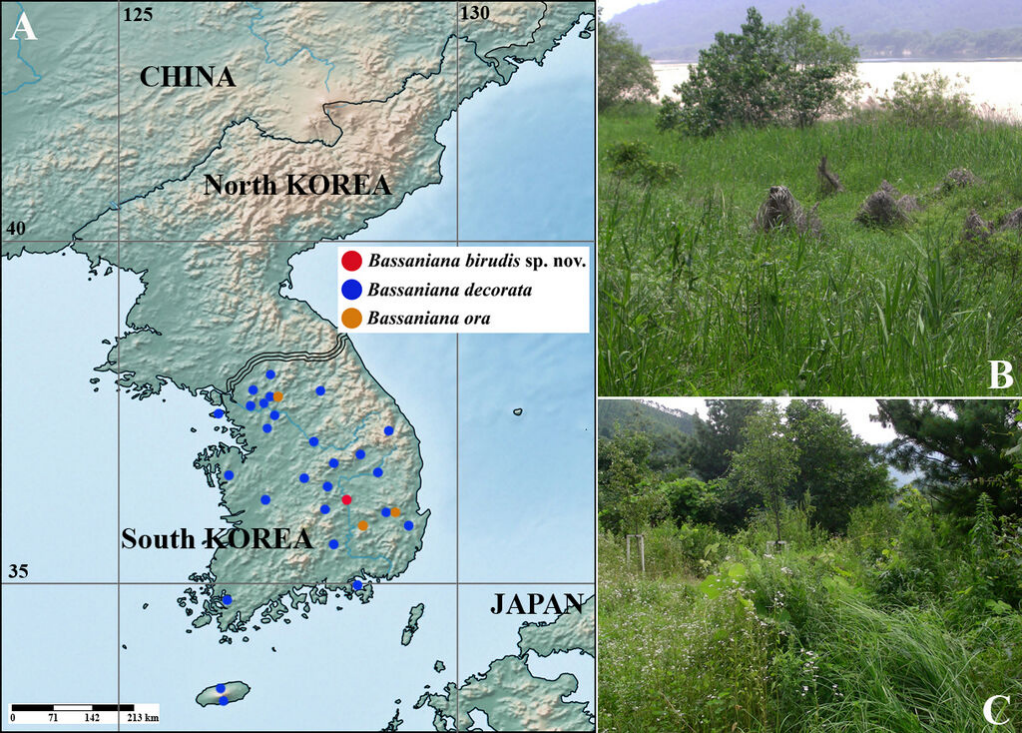
Distribution map and habitat. **A.** Distribution of three species of *Bassaniana* in South Korea; **B, C.** Habitat of *Bassanianabirudis* **sp. nov.**

The genus *Bassaniana* Strand, 1928 in the family Thomisidae Sundevall, 1833 was erected with *Bassanianaversicolor* (Keyserling, 1880) as the type species from North America. This genus is species-poor comprising of only six species currently worldwide (World Spider Catalog 2021). It belongs to the subfamily Thomisinae Sundevall, 1833 within the family, which was taxonomically circumscribed and diagnosed, based on morphology by Ono (1988). The general appearance and male palp of the genus *Bassaniana* closely resemble those of the genera *Coriarachne* Keyserling, 1880, *Xysticus* C. L. Koch, 1835, and *Ozypila* Simon, 1864 within the subfamily. However, *Bassaniana* species can be distinguished from by the lack of extremely flattened body from *Coriarachne* species which has an extremely flattened body, and by the prolaterally curved ventral tibial apophysis and a short embolus from *Xysticus* species which has a various shaped ventral tibial apophysis pointing in different directions and a long embolus. *Bassaniana* species also can be distinguished by the lack of an intermediate tibial apophysis or unbifurcated retrolateral tibial apophysis and a simple bulb without a tegular apophysis from *Ozypila* species which has an intermediate tibial apophysis or a bifurcated retrolateral tibial apophysis and a complex bulb usually having a tegular apophysis (Dondale and Redner 1978, Ono 1988). Two species, *Bassanianadecorata* (Karsch, 1879) and *Bassanianaora* Seo, 1992, are known in South Korea; *B.decorata* is widely distributed throughout South Korea and distributed in Russia, China and Japan, but the Korean endemic *B.ora* is rare with its limited distribution (Fig. 1A) (Kim et al. 2016, Kim 2019, World Spider Catalog 2021). Two males of *Bassanianabirudis* sp. nov. were collected from Gumi-si, Gyeongsangbuk-do (South-central inland area in South Korea) during an intensive seasonal investigation of the spider fauna in hilly provinces nationwide in 2019-2020 (Fig. 1B). The male of *Bassanianabirudis* sp. nov. is described with measurements, a key to the males of Korean *Bassaniana* species, morphological illustrations, and a distribution map.

## Materials and methods

External morphology was examined and illustrated using a stereoscopic dissecting microscope (LEICA, S8APO, Singapore). Images of habitus were taken with a CANON 650D digital camera with 60 mm macro-lens. Measurements of body parts were made with an ocular micrometer and are recorded in millimeters. Leg and palp (left) measurements are given as leg number, total length (femur, patella, tibia, metatarsus, tarsus). Terminology used to describe the palpal characters follows Dondale and Redner (1978)and Ono (1988). Abbreviations used are as follows: ALE = anterior lateral eye, AME = anterior median eye, PLE = posterior lateral eye, PME = posterior median eye, AER = anterior eye row, PER = posterior eye row; RTA = retrolateral tibial apophysis, VTA ventral tibial apophysis.

## Taxon treatments

### Bassaniana birudis sp. n.

72DB8D07-7471-541F-A754-DB224A6BC199

E81A677A-8861-4532-A5D7-3ACDFC3C00C3

#### Materials

- **Type status:** Holotype. **Occurrence:** recordedBy: Jae Seong Im and Seung Tae Kim; individualCount: 1; sex: male; lifeStage: adult; **Taxon:** phylum: Arthropoda; class: Arachnida; order: Araneae; family: Thomisidae; **Location:** continent: Asia; country: South Korea; countryCode: KR; stateProvince: Gyeongsangbuk-do; municipality: Gumi-si; locality: Gupo-dong; decimalLatitude: 36.128139; decimalLongitude: 128.396056; **Identification:** identifiedBy: Seung Tae Kim; **Event:** samplingProtocol: sweep net; eventDate: Jun-25-2020; habitat: mixed forest; **Record Level:** institutionID: Nakdonggang National institute of Biological Resources (NNIBR)
- **Type status:** Paratype. **Occurrence:** recordedBy: Sue Yeon Lee and Seung Tae Kim; individualCount: 1; sex: male; lifeStage: adult; **Taxon:** phylum: Arthropoda; class: Arachnida; order: Araneae; family: Thomisidae; **Location:** continent: Asia; country: South Korea; countryCode: KR; stateProvince: Gyeongsangbuk-do; municipality: Gumi-si; locality: Gupo-dong; decimalLatitude: 36.128139; decimalLongitude: 128.396056; **Identification:** identifiedBy: Seung Tae Kim; **Event:** samplingProtocol: sweep net; eventDate: May-14-2020; habitat: mixed forest; **Record Level:** institutionID: Konkuk University (KKU)

#### Description

**Figure 2.**
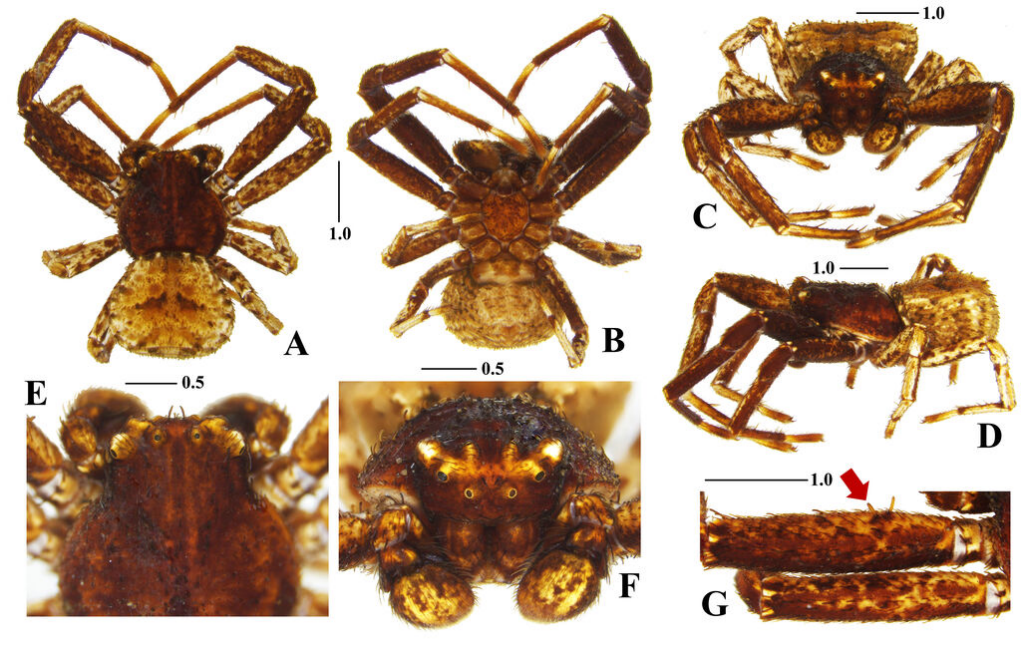
*Bassanianabirudis* **sp. nov.**, holotype male. **A.** Habitus in dorsal view; **B.** Ditto in ventral view; **C.** Ditto in frontal view; **D.** Ditto in lateral view; **E.** Eye area from above; **F.** Ditto from front; **G.** Femur; **I.** in prolateral view (arrow indicates two proximal protuberances). Scale bars in mm.

**Figure 3.**
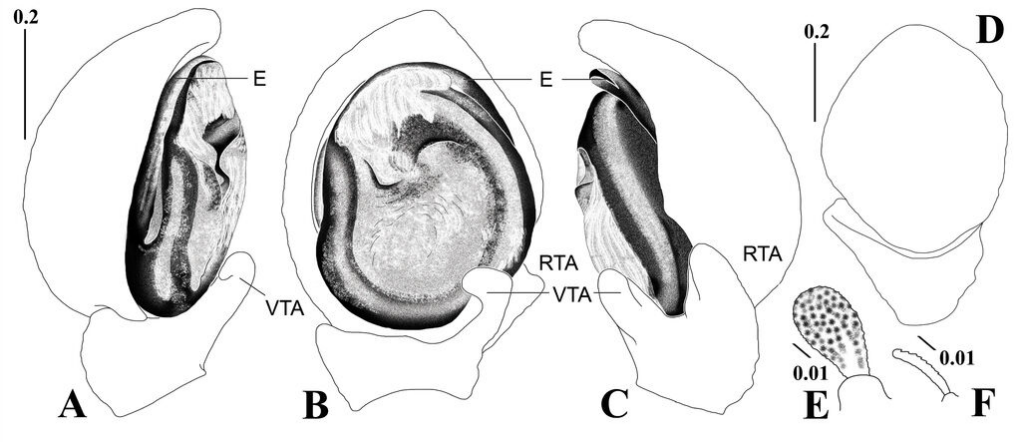
*Bassanianabirudis* **sp. nov.**, holotype male. **A.** Palp in prolateral view; **B.** Ditto in ventral view; **C.** Ditto in retrolateral view; **D.** Ditto in dorsal view; **E.** Clavate seta; **F.** Serrated seta (E, embolus; RTA, retrolateral tibial apophysis; VTA, ventral tibial apophysis). Scale bars in mm.

**Holotype male**. Total length 4.20 (habitus). Carapace: 2.15 long/2.20 wide, dark reddish-brown, round, slightly wider than long, clothed sparsely with serrated setae especially along the cervical furrow, cephalic region of prosoma flat with a pair of light stripes along the median line, numerous warts present, cervical and radial furrows distinct, dark longitudinal fovea slightly depressed (Fig. 2A and, C-E). Eyes: ALE 0.10, AME 0.06, PLE 0.14, PME 0.06, ALE-AME 0.17, AME-AME 0.30, PLE-PME 0.37, PME-PME 0.31, ALE-PLE 0.30, AME-PME 0.37, AER 0.58, PER 0.59, all eyes on the eye tubercle and lateral eye tubercles conspicuously developed, eight eyes in two rows, AER almost straight and PER recurved from above, AER strongly procurved and PER slightly procurved from front, PER longer than AER (Fig. 2E and, F). Chelicera; 0.62 long/0.35 wide, dark reddish-brown, light stripe and cross-shaped pattern on dorsal surface (Fig. 2C), no cheliceral teeth, fang very short. Endite: 0.45 long/0.18 wide, dark reddish-brown. Labium: 0.28 long/ 0.25 wide, dark reddish-brown. Sternum: 1.00 long/0.98 wide, mottled with dark and light reddish-brown, subcordate, clothed sparsely with long blackish-brown setae, pointed anteromedial margin protrudent, posterior end round and not protrudent between the coxae of leg IV (Fig. 2B). Legs: I 7.67 (2.40, 0.80, 1.67, 1.90, 0.90), II 6.84 (2.07, 0.80, 1.50, 1.65, 0.82), III 3.92 (0.87, 0.60, 0.85, 0.93, 0.67), IV 4.40 (1.00, 0.52, 0.90, 1.18, 0.80), ivory, stout and strongly developed, I and II mottled severely with dark reddish-brown, III and IV mottled weakly with dark reddish-brown (Fig. 2A and, D), femur with ventral stripe (Fig. 2B), femur I with two small rod-like proximal protuberances on prolateral surface (Fig. 2G), leg formula I-II-IV-III. Abdomen: 2.10 long/2.03 wide, ivory, flat and mottled with blackish-brown, yellowish-brown and reddish-brown, trapezoidal, slightly longer than wide, a pair of dark reddish-brown triangular markings paramedianly, numerous round or irregular pits on dorsal surface, clothed densely with semi-transparent clavate and serrated setae (Fig. 2A, C and, D; Fig. 3E and, F). Palp: 2.57 (0.46, 0.41, 0.20, - , 0.70), bulb round and simple, left margin of tegulum slightly constricted, no tegulum apophysis, embolus thick with a pointed embolus tip straight rotating largely clockwise and close to the tegulum, thumb-like VTA large with a bent tip, thumb-like blunt RTA large (Fig. 3A–D).

**Female.** Unknown.

#### Diagnosis

The male of the new species can be easily distinguished from congeners of this genus, except *B.ora* by the thumb-like blunt RTA without a terminal spur (Fig. 3B and, C); versus thumb-like RTA with a spine-like terminal spur in *B.baudueri* (Simon, 1877) (Breitling et al. 2016: 44, figs. 8–9), *B.decorata* (Karsch, 1879) (Paik 1974: 120, figs. 5–6), *B.floridana* (Banks, 1896) (Bowling and Sauer 1975: 188, f. 4), *B.utahensis* (Gertsch, 1932) (Dondale and Redner 1978: 136, figs. 439 and, 441), and *B.versicolor* (Dondale and Redner 1978: 139, figs. 443 and, 445). The male of the new species is most similar to *B.ora* in the shape of the palpal organ, but can be easily distinguished from the latter by the body appearance, the shape of VTA, tegulum, and embolus: males of *B.birudis* sp. nov. have no white patterns on the carapace and abdominal dorsum (Fig. 2A), a thick thumb-like VTA (Fig. 3C), a slightly constricted tegulum (Fig. 3B), and an embolus close to the tegulum (Fig. 3B), *versus* white marginal patterns on the carapace and abdominal dorsum, a slender finger-like VTA, a round tegulum, and an embolus separated from the tegulum in *B.ora* (Seo 1992: 79, figs. 1–4).

#### Etymology

The species name is a combination of Latin prefix ‘bi-’ (meaning two) and noun ‘rudis’ (meaning small stick) referring to two small rod-like proximal protuberances on the prolateral surface of the femur I (Fig. 2G).

#### Distribution

South Korea: Gumi-si, Gyeongsangbuk-do (Fig. 1A).

#### Notes

##### Remarks

The species was collected with a sweep net between shrubs in a mixed forest of hilly terrain near the tributary of the Nakdonggang River. Currently, females have not been collected and are not known, and no ecological information is available for this species other than its habitat.

## Identification Keys

### Key to the males of South Korean species of *Bassaniana*

**Table:** 

1	Embolus tip curled, VTA with a spine-like terminal spur	*B.decorata* (Karsch)
–	Embolus tip straight, VTA without terminal spur	2
2	VTA finger-like and slender, tegulum constricted, distinct, embolus separated from the tegulum	*B.ora* (Seo)
–	VTA thumb-like and thick, tegulum unconstricted, embolus close to the tegulum	*B.birudis* sp. nov.

## Supplementary Material

XML Treatment for Bassaniana birudis

## References

[B7385021] Bowling T. A., Sauer R. J. (1975). A taxonomic revision of the crab spider genus *Coriarachne* (Araneida, Thomisidae) for North America north of Mexico.. Journal of Arachnology.

[B7385039] Breitling Rainer, Bauer Tobias, Grabolle Arno, Oger Pierre, Pantini Paolo, Van Keer Johan, Pfliegler W. P., Jantscher Elke, Dolanský J. (2016). East meets West: on the true identity of *Cheiracanthiumrupestre* and *Xysticusalbomaculatus* (Arachnida: Araneae: Eutichuridae, Thomisidae).. Arachnologische Mitteilungen.

[B7385249] Dondale C. D., Redner J. H. (1978). The insects and arachnids of Canada, Part 5. The crab spiders of Canada and Alaska, Araneae: Philodromidae and Thomisidae.. Research Branch Agriculture Canada Publication.

[B7385324] Kim S. T., Lee S. Y., Im M. S., Yoo J. S. (2016). Distribution of Korean spiders..

[B7410808] Kim S. T. (2019). Class Arachnida, order Araneae..

[B7385341] Ono H. (1988). A revisional study of the spider family Thomisidae (Arachnida, Araneae) of Japan..

[B7385453] Paik K. Y. (1974). Korean spiders of genus *Oxyptila* (Araneae, Thomisidae).. Educational Journal of the Teacher's College Kyungpook National University.

[B7385358] Seo B. K. (1992). Descriptions of two species of the family Thomisidae from Korea.. Korean Arachnology.

[B7385418] Catalog World Spider (2021). World spider catalog. Version 22.0.. http://wsc.nmbe.ch.

